# Supplementary material for: Oligo DNA-based quantum dot (QD) single-particle tracking for multicolor single-molecule imaging
Source: Biophys Physicobiol. 2026 Mar 27;23(2):e230013. doi: 10.2142/biophysico.bppb-v23.0013 (PMC13310573; doi:10.2142/biophysico.bppb-v23.0013)
Supplement: Supplementary file 1 — Supplementary Materials [file 23_e230013_1.pdf]

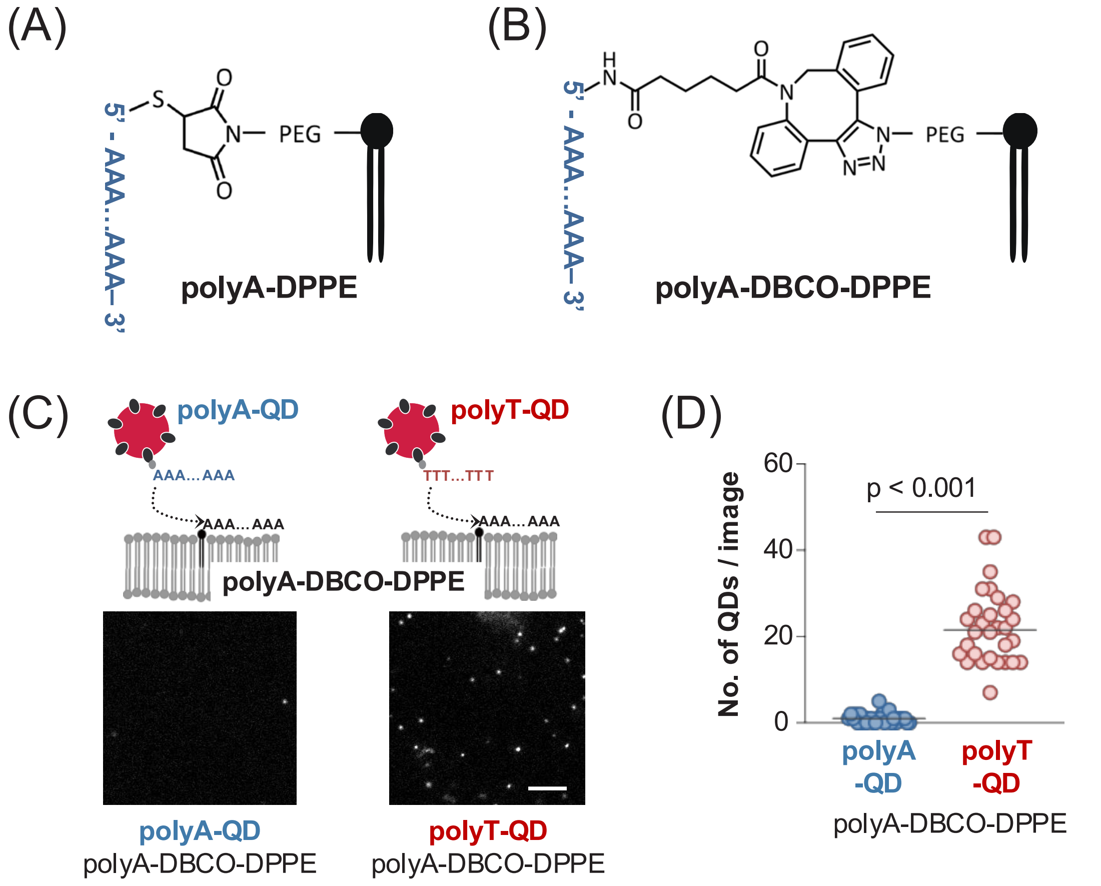

Supplementary Figure S1

Effect of the linker between DPPE and ssDNA on QD-labeling specificity
(A) PolyA–DPPE generated via thiol–maleimide coupling. (B) PolyA–DPPE–DBCO generated via DBCO–azide click chemistry. (C) Representative images of polyA–DPPE–DBCO labeled with polyA–QD (left) or polyT–QD (right). Bar, 10 μm. (D) Number of QDs per field (41.8 × 41.8 μm; solid lines, medians; n = 29 images for polyA–QD and n = 30 images for polyT–QD). Mann–Whitney U test.

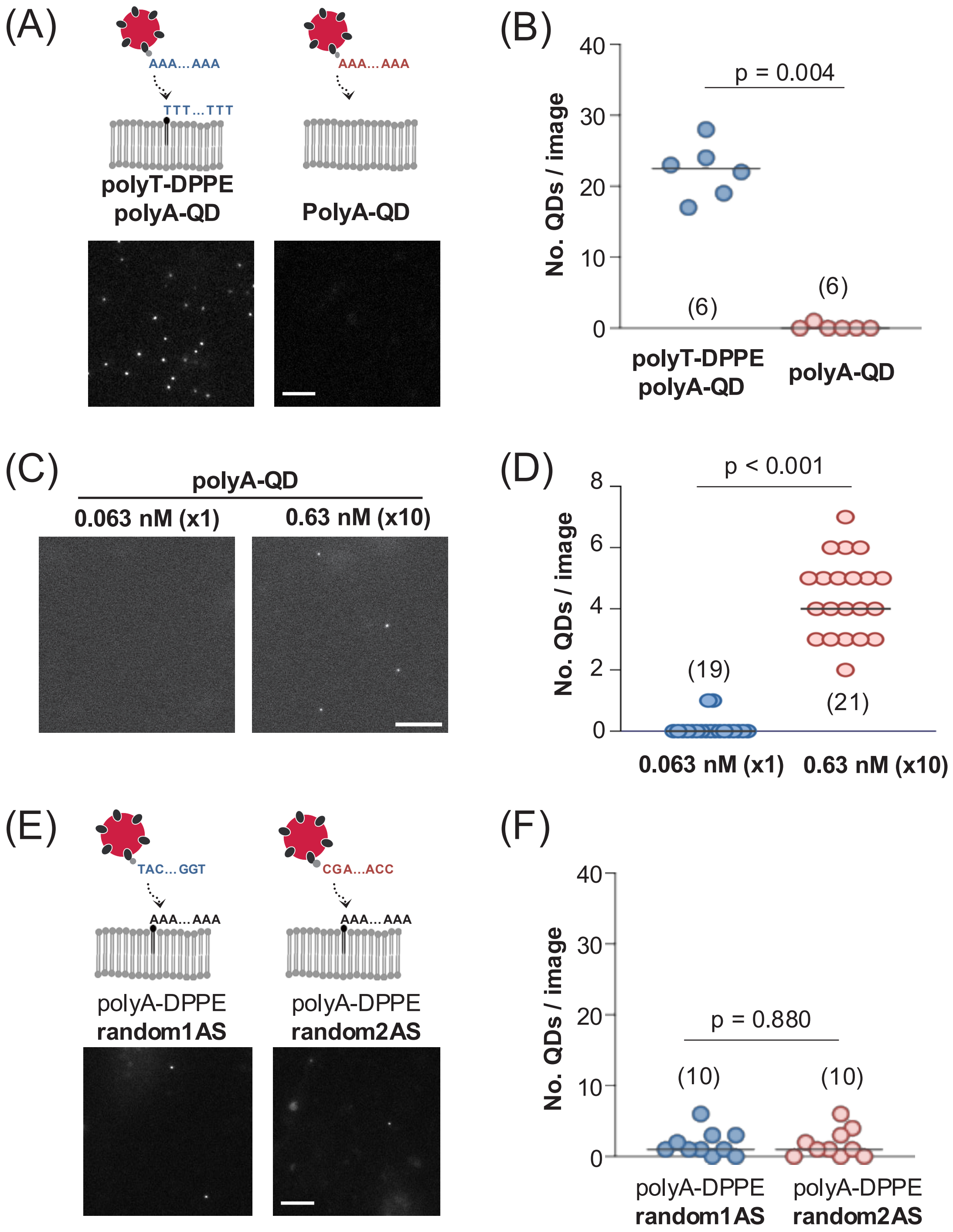

Supplementary Figure S2

Specificity of ssDNA–QD binding to the DPPE

A) Representative images of neurons incubated with polyA–QD in the presence (left) or absence (right) of polyT–DPPE. (B) Number of QDs per field (41.8 × 41.8 µm; solid lines, medians).
(C) Representative images of neurons incubated with 0.063 or 0.63 nM polyA–QD. (D) Number of QDs per field (solid lines, medians). (E) Representative images of neurons expressing polyT–DPPE labeled with QDs carrying random sequences (random1AS or random2AS). (F) Number of QDs per field (solid lines, medians).
Bars in (A), (C), and (E), 10 µm. Statistical analysis: (B, D) Mann–Whitney U test. N is shown in parentheses (see Table S2).

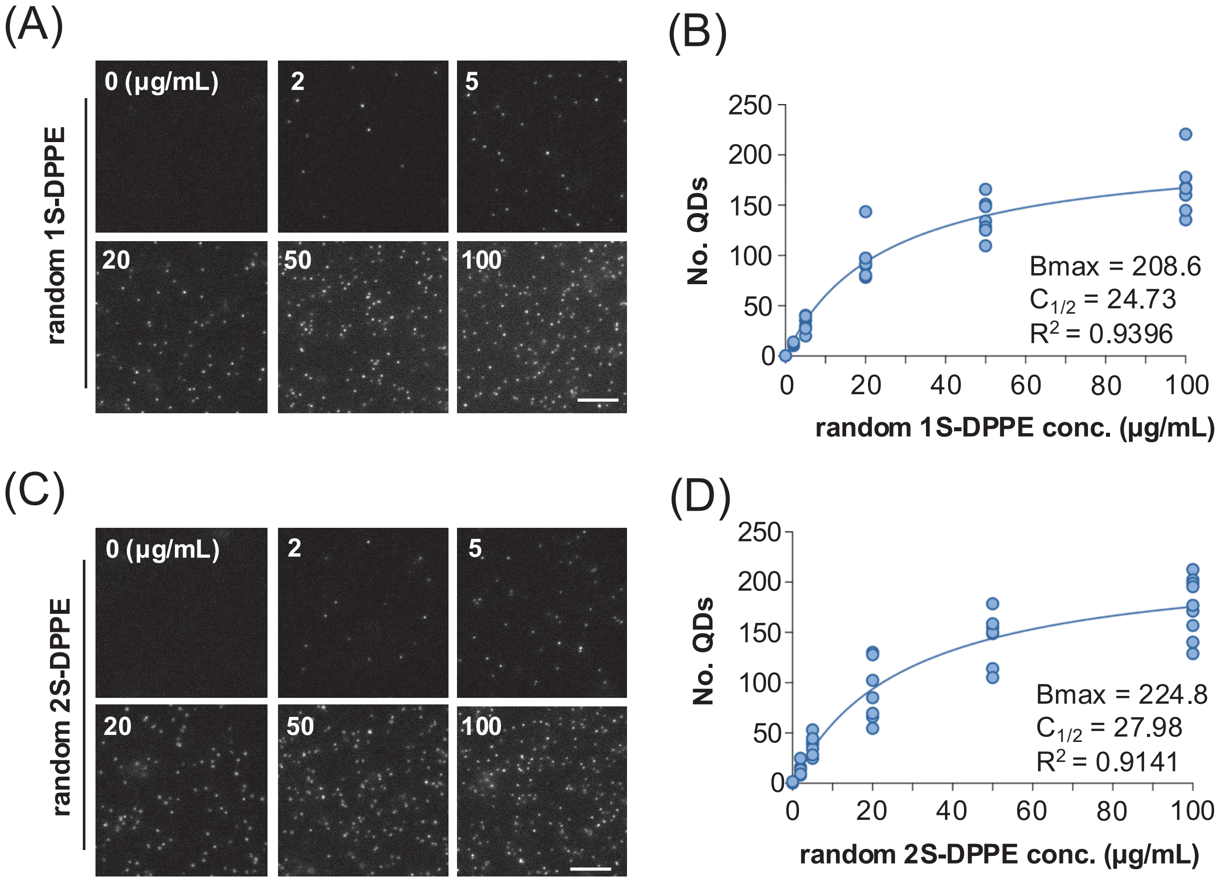

Supplementary Figure S3

Number of QDs at different concentrations of random 1S-DPPE and random 2S-DPPE.
(A, C) Representative QD images for random 1S-DPPE + random 1AS-QD (A) and random 2S-DPPE + random 2AS-QD (C) at the indicated DPPE concentrations (0, 2, 5, 20, 50, 100 µg/mL). Bar, 10 µm.
(B, D) Number of QDs per field of view (41.8 × 41.8 µm) for random 1S-DPPE (B) and random 2S-DPPE (D). Each point represents one field. n = 5 images (0 µg/mL) and 7 images (others) for random 1S-DPPE; n = 5 images (0 µg/mL), 7 images (2–50 µg/mL), and 9 images (100 µg/mL) for random 2S-DPPE.

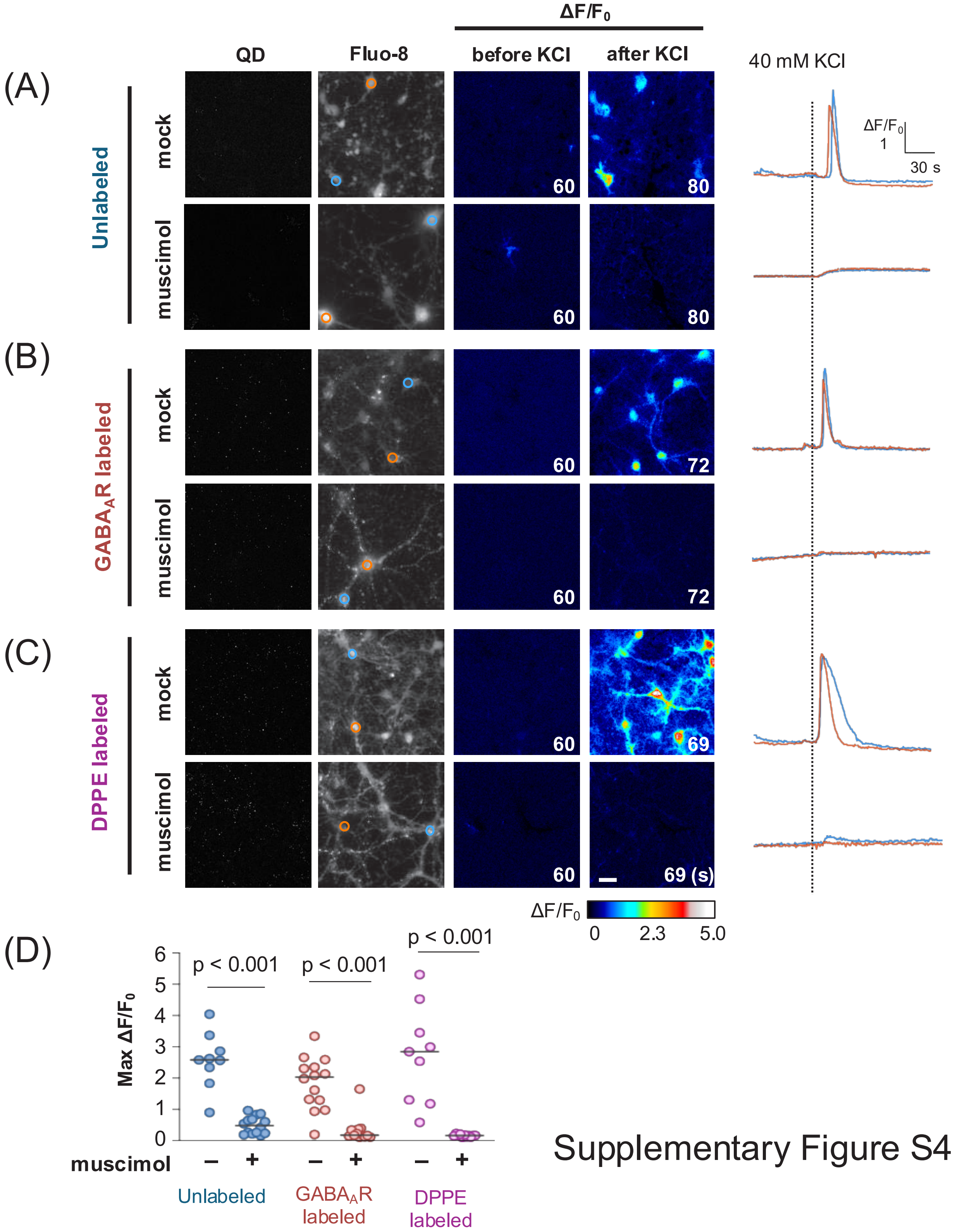

Supplementary Figure S4

Comparison of KCl-induced intracellular Ca^{2+} responses in the presence or absence of muscimol under QD-labeled and unlabeled conditions.

(A–C) QD images, Fluo-8 images, pseudocolor $\Delta F/F_0$ images before and after 40 mM KCl application, and representative $\Delta F/F_0$ traces of neurons with or without preincubation with 20 μM muscimol. Bar, 20 μm. (A) Unlabeled cells; (B) $GABA_AR$-labeled cells; (C) DPPE-labeled cells.

(D) Maximum $\Delta F/F_0$ with (+) or without (–) muscimol under unlabeled, $GABA_AR$-labeled, and DPPE-labeled conditions (solid lines, medians). n = 9 (unlabeled), 14 (unlabeled + muscimol), 14 ($GABA_AR$-labeled), 12 ($GABA_AR$-labeled + muscimol), 9 (DPPE-labeled), and 11 (DPPE-labeled + muscimol). Statistical analysis: Mann–Whitney U test.

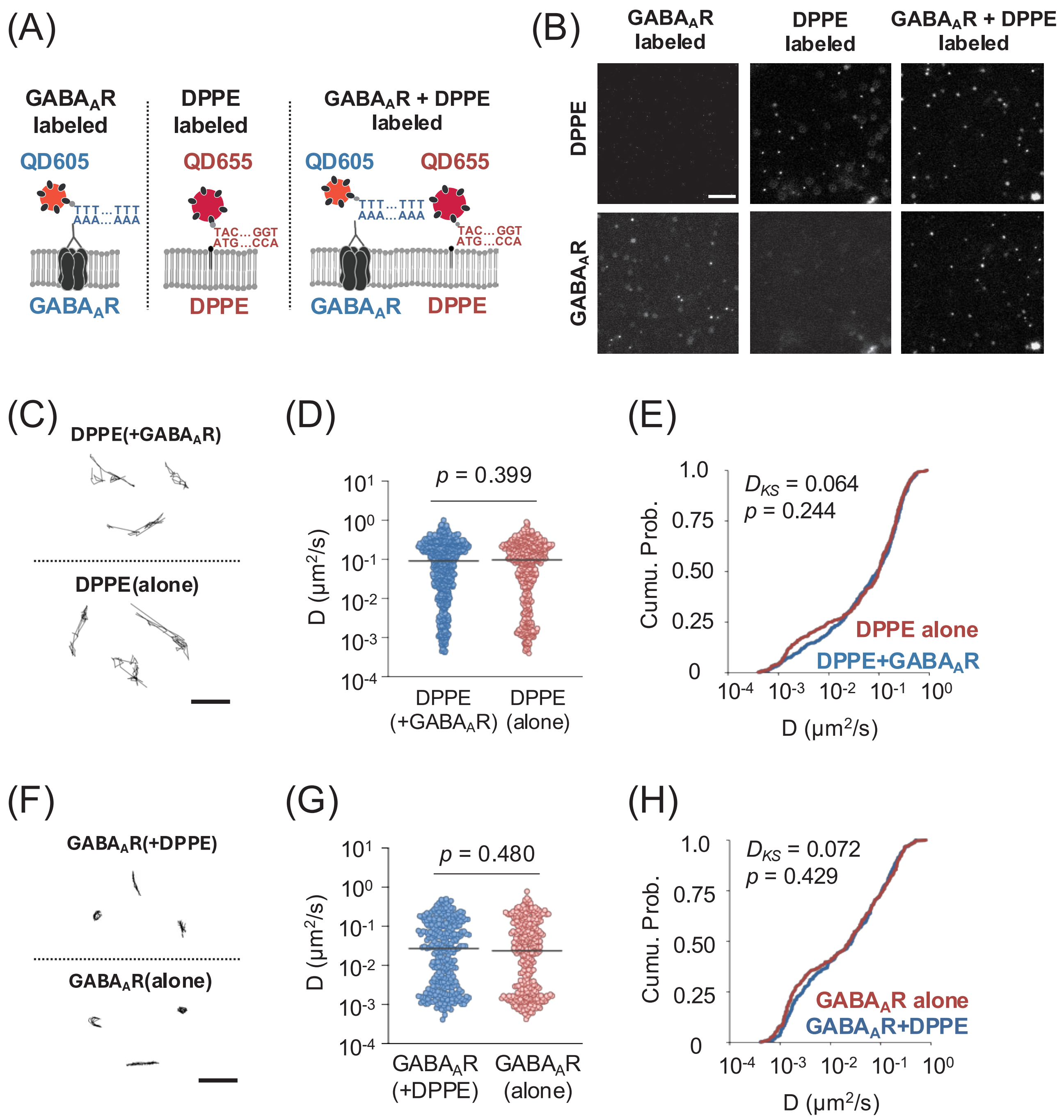

Supplementary Figure S5

Comparison of diffusion coefficients under single-color and multicolor QD-labeling conditions. (A) Strategy for single-color and multicolor QD labeling of $GABA_AR$ (QD605) and DPPE (QD655) using oligoDNAs. (B) Representative QD-labeling images. Bars, 10 μm. (C) Representative trajectories of DPPE under single-color and multicolor labeling. Bar, 1 μm. (D, E) Distribution of D for DPPE shown as a dot plot (D) and cumulative distribution (E) (solid lines, medians). n = 451 (DPPE alone) and 611 (DPPE + $GABA_AR$). (F) Representative trajectories of QD-$GABA_AR$ under single-color and multicolor labeling. Bar, 1 μm. (G, H) Distribution of D for $GABA_AR$ shown as a dot plot (G) and cumulative distribution (H) (solid lines, medians). n = 298 ($GABA_AR$ alone) and 300 ($GABA_AR$ + DPPE). Statistical analysis: (D, G) Mann–Whitney U test; (E, H) Kolmogorov–Smirnov test.

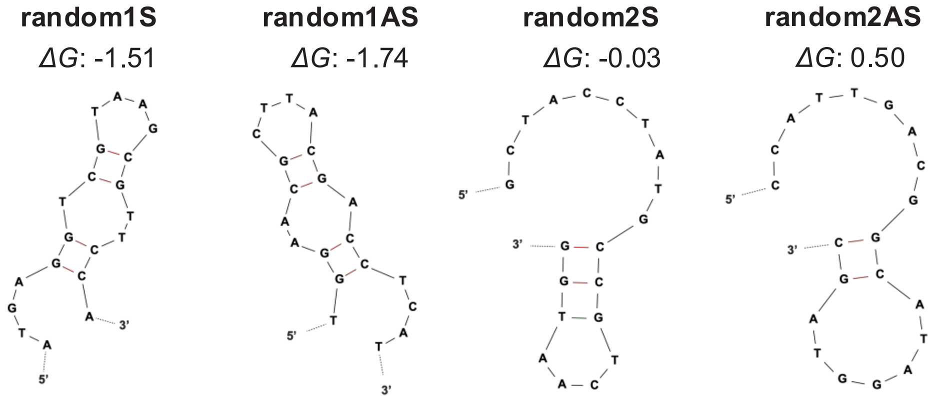

Supplementary Figure S6

Secondary structures predicted by mfold [33] for the random ssDNAs used in this experiment. Red lines indicate canonical base pairs of Watson–Crick type, which contribute significantly to the thermodynamic stability of the structure. Green lines indicate non-canonical or weak base pairing; ΔG denotes free energy change [kcal/mol].

Supplementary Table S1 ssDNA nucleotide sequences.
List of ssDNA sequences used and melting temperatures (Tm) estimated by nearest neighbor base pairing (Breslauer et al., 1986). PolyT and PolyA, random1S and 1AS, random2S and 2AS, are complementary pairs.

ssDNA name	Sequence	Tm (°C)
polyA	5’-AAAAAAAAAAAAAAAAAAAA-3’	52.17
polyT	5’-TTTTTTTTTTTTTTTTTTTT-3’	52.17
Random1S	5’-ATGAGGTCGTAAGCGTTCCA-3’	64.47
Random1AS	5’-TGGAACGCTTACGACCTCAT-3’	64.47
Random2S	5’-GCTACCTATGCCGTCAATGG-3’	64.21
Random2AS	5’-CCATTGACGGCATAGGTAGC-3’	64.21

Supplementary Table S2: Summary of the median and Interquartile Range (IQR) for the data

The median and IQR of each data sets are shown here.

Figure	group	Median (spots/image)	IQR (25% – 75%) (spots/image)	N(images)
**Figure 2C**	polyA-QD	1	0 – 2	10
	polyT-QD	241	96 – 400	10
**Figure 2F**	polyA	3	2 – 4	10
	polyT	3	2 – 4	10
	random1AS	40	26 – 49	10
	random2AS	1	0 – 2	10
**Figure 2I**	mock (0min)	25	17 – 29	11
	mock (3min)	27	20 – 30	
	DNase (0min)	31	24 – 33	11
	DNase (3min)	10	4 – 11	
**Figure 4C**	polyA-polyT	193	169 – 206	5
	random1S-1AS	20	17 – 25	5
**Figure 5C**	polyA-QD	2	1 – 3	30
	polyT-QD	28	22 – 44	30
**Figure S1D**	polyA-QD	1	0 – 1	29
	polyT-QD	22	15 – 26	30
**Figure S2B**	polyT-DPPE + polyA-QD	23	20 – 24	6
	polyA-QD	0	0 – 0	6
**Figure S2D**	0.063 nM	0	0 – 0	19
	0.63 nM	4	3 – 5	21
**Figure S2F**	random1AS	1	1 – 3	10
	random2AS	1	0 – 3	10

Figure	group	Median (μm²/s)	IQR (25% – 75%) (μm²/s)	N
**Figure 3C**	glass attached	2.40×10^{-2}	$1.66 – 3.03 \times 10^{-2}$	152 spots
**3D**	lower	1.15×10^{-1}	$5.70 – 26.6 \times 10^{-2}$	368 spots
	upper	2.21×10^{-1}	$1.26 – 3.43 \times 10^{-1}$	584 spots
**Figure 3H**	lower	8.23×10^{-2}	$0.387 – 20.6 \times 10^{-2}$	879 QDs
**3I**	upper	1.86×10^{-1}	$9.13 – 31.5 \times 10^{-2}$	815 QDs
**Figure 4E**	polyA-polyT	1.38×10^{-1}	$7.74 – 22.6 \times 10^{-2}$	219 QDs
**4F**	random1S-1AS	1.47×10^{-1}	$7.86 – 23.0 \times 10^{-2}$	253 QDs
**Figure 5F**	ssDNA	6.49×10^{-2}	$1.41 – 12.3 \times 10^{-2}$	969 QDs
**5G**	Fab	5.19×10^{-2}	$0.841 – 12.8 \times 10^{-2}$	686 QDs
**Figure 6D**	$GABA_AR$	2.32×10^{-2}	$0.300 – 9.56 \times 10^{-2}$	419 QDs
**6E**	DPPE	1.15×10^{-1}	$0.826 – 24.5 \times 10^{-2}$	412 QDs
**Figure S6D**	DPPE (alone)	9.74×10^{-2}	$1.14 – 21.6 \times 10^{-2}$	451 QDs
**S6E**	DPPE (+$GABA_AR$)	9.18×10^{-2}	$1.78 – 22.9 \times 10^{-2}$	611 QDs
**Figure S6G**	$GABA_AR$ (alone)	2.38×10^{-2}	$0.19 – 11.1 \times 10^{-2}$	298 QDs
**S6H**	$GABA_AR$ (+DPPE)	2.72×10^{-2}	$0.29 – 10.5 \times 10^{-2}$	300 QDs

Figure	group	Median (%)	IQR (25% – 75%) (%)	N (images)
**Figure 3G**	lower	17.3	4.53 – 25.1	28
	upper	2.16	0 – 7.80	28

Figure	group	Median (max $\Delta F/F_0$)	IQR (25% – 75%) (max $\Delta F/F_0$)	N (images)
**Figure S4D**	Unlabeled (–muscimol)	2.58	2.34 – 2.86	9
	Unlabeled (+muscimol)	0.48	0.24 – 0.67	14
	$GABA_A R$ (–muscimol)	2.03	1.30 – 2.34	14
	$GABA_A R$ (+muscimol)	0.18	0.14 – 0.35	12
	DPPE (–muscimol)	2.84	1.30 – 3.45	9
	DPPE (+muscimol)	0.16	0.13 – 0.18	11
